# Gender differences in sudden cardiac death in the young-a nationwide study

**DOI:** 10.1186/s12872-016-0446-5

**Published:** 2017-01-07

**Authors:** Bo Gregers Winkel, Bjarke Risgaard, Thea Bjune, Reza Jabbari, Thomas Hadberg Lynge, Charlotte Glinge, Henning Bundgaard, Stig Haunsø, Jacob Tfelt-Hansen

**Affiliations:** Department of Cardiology, Copenhagen University Hospital, Rigshospitalet, 2142, Blegdamsvej 9, 2100 Copenhagen O, Denmark

**Keywords:** Sudden cardiac death, Sudden death, SCD, Epidemiology, Young, Registries, Gender, Causes of death, Comorbidity

## Abstract

**Background:**

Hitherto, sudden cardiac death (SCD) in the young has been described with no distinction between genders. SCD occurs more often in men (SCDm) than women (SCDw), but this disparity is not understood and has not been investigated systematically in a nationwide setting. Our objective was to report gender differences in SCD in the young in a nationwide (Denmark) setting.

**Methods:**

All deaths in persons aged 1–35 years nationwide in Denmark between 2000 and 2009 were included. Death certificates and autopsy reports were obtained. The extensive health care registries in Denmark were used to investigate any known disease prior to death. SCDw were compared to SCDm.

**Results:**

During the 10-year study period there were a total of 8756 deaths in 23.7 million person-years. In total, 635 deaths were SCD. SCDw constituted 205 deaths (32%). Women had a higher proportion of witnessed deaths (51 vs. 41%, *p* = 0.02) and died less often in a public place (16 vs. 26%, *p* = 0.01). Age at death, ratios of autopsies and sudden unexplained deaths, and comorbidities, did not differ. Causes of SCD were largely comparable between genders. The incidence rate of SCDw was half of that of SCDm (1.8 vs. 3.6 per 100,000 person-years, incidence rate ratio 2.0 (95% CI 1.7–2.4), *p* < 0.01).

**Conclusions:**

Incidence rate ratio of SCDm vs SCDw is 2. Young SCDw and SCDm are equally investigated, have comparable comorbidity, and causes of SCD. SCD due to potentially inherited cardiac diseases is less often in young women and could reflect a protection of female gender.

## Background

Sudden cardiac death (SCD) has been given great attention over the past two decades. Despite multiple approaches for treatment and prophylactic measures, SCD remains a major health problem, accounting for 50–70% of all cardiovascular deaths in the developed countries [1–6].

Although the incidence of SCD increases with age in both genders [7, 8], the annual rate of SCD in women (SCDw) is almost half of that of men [8, 9]. As a consequence, studies describing SCD mainly represents findings in men, making the interpretation of the pathophysiology of SCDw less certain. In general, the underlying causes of SCD is believed to be of similar nature in both genders, still the limited amount of data available suggests that differences may exist [10–12]. Studies have also shown that SCDw is more likely to occur in the absence of prior overt coronary artery disease (CAD) and that women are somewhat more prone to have unrecognized myocardial infarction [8, 11, 13, 14]. This might indicate that SCD among women may be more difficult to predict and prevent.

Even though SCD in children and adolescents is an uncommon event, it makes up a significant proportion of the mortality [15–20], accounting for 7% of all deaths in the 1–35 years old [20]. Despite an increased risk of SCD with increasing age, the proportion of cardiac deaths being sudden declines [21]. The tragic incident of SCD has great impact on the community and the families involved, especially taking into account that SCD commonly occur in apparently healthy, young individuals. As a result, great effort has been put into investigating the underlying causes of death associated with SCD in the young [17, 19, 20, 22, 23]. Few studies have focused on gender in SCD and in cardiac arrest (CA) survivors in older selected populations [11, 21, 24]. As such, gender differences in the young have never been investigated, and the huge difference in incidence rates among gender in young SCD is not understood. The possibility that young women below the age of 35 years exert a different SCD risk profile merits consideration. We have previously identified all SCD in young Danes aged 1–35 years between 2000 and 2009 [19, 20]. In a nationwide setting (Denmark) we systematically investigated SCDw and analyzed gender differences. We aimed to report causes of SCDw, as well as autopsy rates and known diseases prior to death.

## Methods

### Study design and population

All deaths in persons aged 1–35 years in Denmark in 2000–2009 were included. The method has previously been described in detail [19, 20]. In brief, all death certificates were retrieved as electronically scanned.tif files. Death certificates were read independently by two physicians to identify cases of sudden death (SD). Autopsy reports on all SDs were collected and read, and cause of death was determined based on autopsy findings. In case of uncertainty regarding the cause of death, the entire case and all its content was reviewed by and discussed with a forensic pathologist. Data on SCD with no distinction on gender has previously been described [19, 20]. The present substudy scrutinizes gender differences in SCD.

The study was approved by the local Ethics Committee (KF 01272484), the Danish Data Protection Agency (2005–41–5237), and the Danish National Board of Health (7–505–29–58/1–5).

### Death certificate data

When a person dies within Danish borders, a death certificate is issued by a medical doctor (physician). In cases where a person is found dead and/or the death was sudden and unexpected, a medico-legal external investigation is mandatory, including a standardized death scene investigation. This investigation is supplemented with data from the hospital records, interviews with the relatives and witnesses, and an external examination of the body conducted by a certified physician.

The Danish death certificate contains a supplemental information field that in detail describes the circumstances relating to the death. Thus, the Danish death certificates can be used as a primary screening tool for sudden unexpected deaths [20].

### Danish national patient registry (NPR)

All Danish citizens have a unique and personal civil registration number that can be linked to national registries on an individual level. The Danish NPR contains information on all in- and outpatient activities in Denmark since 1978 [25]. All contacts are registered with timestamps, hospital department, type of contact, and ICD-10 diagnosis.

### Conduction of autopsies

In Denmark, forensic autopsies are performed in cases where the external examination warrants it. All autopsies are supervised by another forensic pathologist. Forensic autopsies follow a standardized protocol, in which all organs are examined. Histopathology is routinely conducted and toxicology screens are performed if considered relevant, i.e., in most cases of sudden unexpected death. In cases where the police do not request an autopsy, or where an external examination was not performed, hospital autopsies may be conducted at the local hospital pathology department, at the request of the relatives and the physician.

### Definitions

We defined SCD in autopsied cases as the sudden natural unexpected death of unknown or cardiac causes; in unwitnessed cases as a person last seen alive and functioning normally, 24 h before being found dead, and in witnessed cases as an acute change in cardiovascular status with the time to death being < 1 h [19, 20].

### Statistical methods

Data are presented as summary data with use of percentages and incidence rates based on population sizes derived from Statistics Denmark [26]. Categorized nominal data were compared using the chi-square test. If any expected cell values were <5, Fisher’s exact test was used. Medians were compared using the Wilcoxon rank-sum test. A two-tailed *p* value <0.05 was considered statistically significant.

## Results

**Fig. 1:**
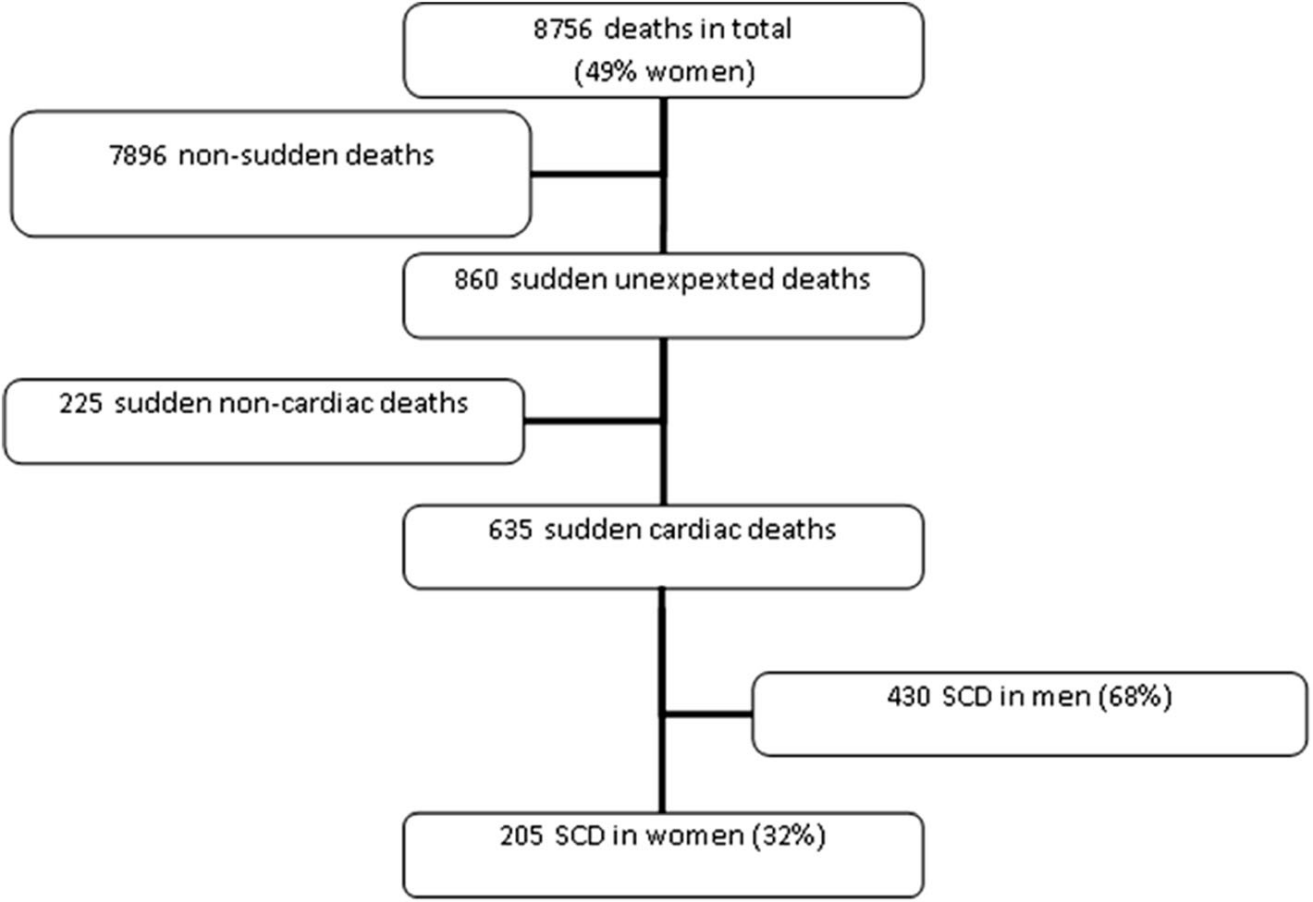
Flowchart of the review of all death certificates and autopsy reports from deceased persons aged 1–35 years between 2000 and 2009 in Denmark

During the 10-year study period there was an average of 2.37 million persons aged 1–35 years, 49% of which were women. There were a total of 8756 deaths from 23.7 million person-years. Of these, 10% (*n* = 860) were sudden unexpected deaths. In total 522 (82%) had an external examination and the autopsy rate was 68%. Of the 860 sudden unexpected deaths, 635 (74%) deaths were SCD, of which SCDw constituted 205 deaths (32%) (Fig. 1). Demographics are shown in Table 1.

**Table 1:** Demographics of SCD in 1–35 years old including gender differences in Denmark 2000–2009

SCD population aged 1–35 years	All *n* = 635 (%)	Men *n* = 430 (%)	Women *n* = 205 (%)	*p*-value
Median age, years	29 (IQR 22–33)	29 (IQR 23–33)	28 (IQR 21–33)	0.19
Witnessed deaths (*n* = 580)	254 (44)	159 (41)	95 (51)	0.02
External examinations	522 (82)	361 (84)	161 (79)	0.10
*Autopsied SCD*	431 (68)	296 (69)	135 (66)	0.45
Explained sudden cardiac death	239 (55)	172 (58)	67 (50)	0.11
Sudden unexplained death	192 (45)	124 (42)	68 (50)	
*Place of death*	(*n* = 627)	(*n* = 426)	(*n* = 201)	
Home	386 (62)	258 (61)	128 (64)	0.45
Public place	144 (23)	111 (26)	33 (16)	0.01
At hospital	74 (12)	44 (10)	30 (15)	0.10
Other	23 (4)	13 (3)	10 (5)	0.23
*Age group 1–18 years*	All *n* = 119 (%)	Men *n* = 77 (%)	Women *n* = 42 (%)	*p*-value
Median age, years	13 (IQR 4–16)	13 (IQR 4–16)	12 (IQR 6–16)	0.72
Witnessed deaths, *n* = 113	62 (55)	36 (49)	26 (67)	0.07
External examinations	101 (85)	70 (91)	31 (74)	0.01
*Autopsied SCD*	88 (74)	62 (81)	26 (62)	0.03
Explained sudden cardiac death	55 (63)	37 (60)	18 (69)	0.40
Sudden unexplained death	33 (38)	25 (40)	8 (31)	
*Place of death*	*n* = 118 (%)	*n* = 77 (%)	*n* = 41 (%)	
Home	65 (55)	42 (55)	23 (56)	0.87
Public place	32 (27)	23 (30)	9 (22)	0.36
At hospital	18 (15)	12 (16)	6 (15)	0.89
Other	3 (3)	-	3 (7)	0.02
*Age group 19–35 years*	All *n* = 516 (%)	Men *n* = 353 (%)	Women *n* = 163 (%)	*p*-value
Median age, years	30 (IQR 27–33)	31 (IQR 27–33)	30 (IQR 25–34)	0.27
Witnessed deaths, *n* = 467	192 (41)	123 (39)	69 (46)	0.12
External examinations	421 (82)	291 (82)	130 (80)	0.47
*Autopsied SCD*	343 (66)	234 (66)	109 (67)	0.90
Explained sudden cardiac death	184 (54)	135 (58)	49 (45)	0.03
Sudden unexplained death	159 (46)	99 (42)	60 (55)	
*Place of death*	*n* = 509	*n* = 349	*n* = 160	
Home	321 (63)	216 (62)	105 (66)	0.42
Public place	112 (22)	88 (25)	24 (15)	0.01
At hospital	56 (11)	32 (9)	24 (15)	0.05
Other	20 (4)	13 (4)	7 (4)	0.73

We found no difference in relation to median age at death: 29 (IQR: 23–33) years for males and 28 (IQR: 21–33) for females, *p* = 0.19 in the age group 1–35 years (Table 1). The proportion of witnessed deaths was higher among women (51 vs. 41%, *p* = 0.02). Compared to SCDm, women less often died in a public place (16 vs. 26%, *p* = 0.01). Activity at time of death was unevenly distributed among men and women (*p* = 0.04) (Table 2). This finding was largely driven by the subgroup aged 19–35 years (*p* = 0.04). In the age group 1–18 years, no such difference in terms of activity at time of death between genders was observed (*p* = 0.35).

**Table 2:** Activity at time of death in SCD victims aged 1–35 years in Denmark 2000–2009, divided by gender

SCD population aged 1–35 years	All *n* = 635 (%)	Men *n* = 430 (%)	Women *n* = 205 (%)	*p*-value
*Activity at death, n* = *544*		*n* = 371	*n* = 173	
Awake and relaxed	285 (52)	193 (52)	92 (53)	0.04
Sleeping	193 (35)	124 (33)	69 (40)	
Moderate to high intensity activity including sport	45 (8)	39 (11)	6 (3)	
Sex	6 (1)	3 (1)	3 (2)	
Eating	6 (1)	4 (1)	2 (1)	
Walking	4 (1)	3 (1)	1 (1)	
Other	5 (1)	5 (1)	-	
*Age group 1–18 years*	All *n* = 119 (%)	Men *n* = 77 (%)	Women *n* = 42 (%)	*p*-value
*Activity at death, n* = *105*		*n* = 71	*n* = 34	
Awake and relaxed	48 (46)	30 (42)	18 (53)	0.35
Sleeping	39 (37)	27 (38)	12 (35)	
Moderate to high intensity activity including sport	16 (15)	13 (18)	3 (9)	
Sex	-	-	-	
Eating	-	-	-	
Walking	1 (1)	-	1 (3)	
Other	1 (1)	1 (1)	-	
*Age group 19–35 years*	All *n* = 516 (%)	Men *n* = 353 (%)	Women *n* = 163 (%)	*p*-value
*Activity at death, n* = *439*		*n* = 300	*n* = 139	
Awake and relaxed	237 (54)	163 (54)	74 (53)	0.04
Sleeping	154 (35)	97 (32)	57 (41)	
Moderate to high intensity activity including sport	29 (7)	26 (9)	3 (2)	
Sex	6 (1)	3 (1)	3 (2)	
Eating	6 (1)	4 (1)	2 (1)	
Walking	3 (1)	3 (1)	-	
Other	4 (1)	4 (1)	-	

There were no difference between genders in the age group 1–35 years in regard to ratio of autopsies and unexplained deaths after autopsy (sudden arrhythmic deaths, SADS) (Table 1). In the subgroup of SCD in children (1–18 years), external examination was more likely performed on males (91 vs. 74%, *p* = 0.01) and women were less often autopsied (62 vs. 81%, *p* = 0.03).

**Fig. 2:**
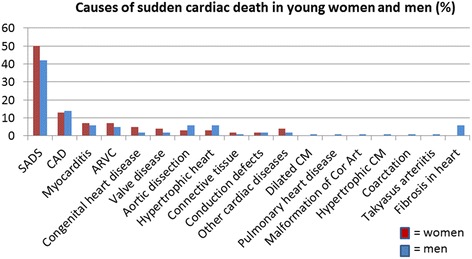
Causes of SCD in young women and men (aged 1–35 years) in percent of total SCD in same gender in 2000–2009 in Denmark. SADS: Sudden arrhythmic death syndrome (unexplained after autopsy), CAD: coronary artery disease, ARVC: Arrhythmogenic right ventricular cardiomyopathy, CM: cardiomyopathy, Cor Art: coronary arteries

Causes of SCDw and SCDm are shown in Table 3 and Fig. 2. Cause of death remained unexplained after autopsy in 50% (*n* = 68) of the female cases. Of the explained SCDw cases, most common structural heart disease was CAD (*n* = 17, 13% of autopsied SCDw), followed by myocarditis and ARVC (*n* = 9 each, 7% of autopsied SCDw). Causes of SCDw were largely comparable to causes of SCDm.

**Table 3:** Causes of sudden cardiac death and potential inherited cardiac disease in SCD victims aged 1–35 years in Denmark 2000–2009, divided by gender

Cause of sudden cardiac death	Men *n* = 296 (%)	Women *n* = 135 (%)	Potential inherited cardiac disease
SADS	124 (42)	68 (50)	X
CAD	41 (14)	17 (13)	X
Myocarditis	19 (6)	9 (7)	
ARVC	16 (5)	9 (7)	X
Congenital heart	6 (2)	7 (5)	
Valve disease	5 (2)	5 (4)	
Aortic dissection	17 (6)	4 (3)	X
Hypertrophic heart	19 (6)	4 (3)	X
Connective tissue	2 (1)	3 (2)	X
Conduction defects	6 (2)	3 (2)	X
Other cardiac diseases	5 (2)	6 (4)	
Dilated CM	4 (1)	-	X
Pulmonary cardiac disease	3 (1)	-	
Malformation of coronary artery	3 (1)	-	
Hypertrophic CM	4 (1)	-	X
Coarctation	2 (1)	-	X
Takyasus	1 (1)	-	
Fibrosis in heart	19 (6)	-	X
Potential inherited cardiac disease	254 (86)	108 (80)

The incidence rate of SCDw was half of that of SCDm among the 1–35 years old (1.8 vs. 3.6 per 100,000 person-years, incidence rate ratio 2.0 (95% CI 1.7–2.4), *p* < 0.01) (Table 4). Similar trends were observed when the population was divided into subgroups.

**Table 4:** Incidence rates of SCD in 1–35 years old, divided by gender

SCD incidence rates per 100.000 person years	All	Men	Women	Incidence rate ratio	*p*-value
1–35 years	2.7 (2.5–2.9)	3.6 (3.2–3.9)	1.8 (1,5–2,0)	2.0 (1.7–2.4)	<0.01
1–18 years	1.0 (0.9–1.2)	1.3 (1.0–1.6)	0.7 (0.5–1.0)	1.7 (1.2–2.6)	0.03
19–35 years	4.4 (3.9–4.8)	5.9 (5.3–6.5)	2.8 (2.4–3.2)	2.1 (1.8–2.6)	<0.01

There were no differences in terms of comorbidity in SCDw and SCDm (Table 5). No significant difference was found between genders regarding known medical history, prior heart disease or congenital heart disease. In total 119 (58%) of the SCDw had a prior medical history, 44 (21%) was due to a known heart disease and 22 (11%) were due to congenital heart disease.

**Table 5:** Comorbidity in SCD victims aged 1–35 years in Denmark 2000–2009, divided by gender

SCD population aged 1–35 years	All *n* = 635 (%)	Men *n* = 430 (%)	Women *n* = 205 (%)	*p*-value
No medical history	275 (43)	189 (44)	86 (42)	0.63
Known heart disease	127 (20)	83 (19)	44 (21)	0.52
Known congenital heart disease	56 (9)	34 (8)	22 (11)	0.24
*Age group 1–18 years*	All (*n* = 119)	Men (*n* = 77)	Women (*n* = 42)	*p*-value
No medical history	55 (46)	37 (48)	18 (43)	0.59
Known heart disease	41 (34)	26 (34)	15 (36)	0.83
Known congenital heart disease	27 (23)	15 (19)	12 (29)	0.26
*Age group 19–35 years*	All (*n* = 516)	Men (*n* = 353)	Women (*n* = 163)	*p*-value
No medical history	220 (43)	152 (43)	68 (42)	0.77
Known heart disease	86 (17)	57 (16)	29 (18)	0.64
Known congenital heart disease	29 (6)	19 (5)	10 (6)	0.73

## Discussion

In this nationwide cohort, the incidence of SCDw was only half compared to men, and women had a higher rate of witnessed deaths. We found no difference in regard to age at death. SCDw more often happened at home and during sleep, and less often in public places and during moderate to high intensity activity. The ratios of external examinations and autopsies were overall similar between genders. In summary we found no additional characteristics to be associated with female gender and both genders had an equal spread of cardiac comorbidity.

Previous studies focusing on older age groups report that men die at younger ages and have a higher prevalence of CAD [1, 8, 10, 27]. As expected, we found an overall increase in SCD rates in both genders with increasing age [7, 8], yet incidence rates of SCDm was twice as high compared to SCDw in all age groups. Our findings imply that SCD manifests more commonly in men, also at younger ages. Of interest, the distribution of causes of death was largely comparable between genders, even though potentially inherited cardiac disease accounts for more than 75% of SCD (see Table 3 and Fig. 2). Even though we must assume that an even distribution of genetic predisposition exists between genders, it seems as if young women die half as often from potentially inherited cardiac diseases than men in the same age group. Even though small in absolute numbers, there was a male dominance in fibrosis (6 vs. 0%). We have no explanation for this finding. Fibrosis in the heart with no ischemic heart disease may be early manifestations of cardiomyopathies. However, in our series this remains speculative, as patients did not fulfill diagnostic criteria for any of the known inherited or acquired cardiomyopathies at time of death/after autopsy.

Place of death varied between genders. Analogous to other studies, we found that women less often died in a public place [28, 29]. The disparity in place of death may partly be explained by the divergence in activity at time of death; men more often died during moderate to high intensity activity and women more often died during sleep. The observed difference in death during activity is well described. In previous studies on sports related SCD there is an overwhelming male predominance [30, 31], although there is no causal explanation to this phenomenon. It is well-known, however, that deaths associated with sport is different than for the population as a whole [30, 31].

The variance in place of death has also been previously thought to reflect why men have a higher frequency of witnessed cardiac arrests [28, 29]. Although our findings on place of death are consistent with previous studies, we found that women aged 1–35 years had a higher proportion of witnessed arrests compared to men. One explanation to this difference could be that other studies investigating SCD were carried out on more elderly population, were women are more likely to live alone thereby contributing to a greater proportion of the unwitnessed arrests [26]. In the contexts of our study population, a higher proportion of young males live alone when compared to the age-matched female population [26].

Whenever a person is found dead in Denmark, and/or the death is sudden and unexpected a standardized thorough medico-legal external examination is mandatory by law. In the present study an external examination was carried out in 82% of all SCD cases and the autopsy ratio was 68%. Of note, the supplemental information field was used in the vast majority of deaths, even in cases were an external examination was not carried out. Some geographical differences in this regard exist, and have previously been thoroughly described [32]. We did not find any gender differences in the age group 1–35 years, regarding neither the ratios of external examinations or autopsies performed. In the subgroup analysis of the 1–18 years old, male gender had a significantly higher rate of both external examinations and autopsies, despite an even distribution of comorbidities. No measurable factors within the study (place of death, activity, previous medical history, known congenital heart disease) can explain this finding, which might just be a spur finding. In absolute numbers, women did have more congenital heart disease. Even though this did not meet statistical significance, it could contribute to the lower autopsy ratio in females 1–18 years of age.

Like previous studies on SCD in younger age groups [22], SADS was frequently concluded after autopsy. Previous studies have shown that women have relatively higher rates of SADS [33, 34]. There was no difference between genders in the age group 1–35 years regarding explained vs. unexplained SCD in the present study. In a population of CA survivors with preserved ejection fraction (CASPER study), with a mean age of 44 years, there were no differences in the percentages of males and females receiving a diagnosis during follow-up, further underscoring the pattern that causes of death, and CA are evenly distributed amongst genders [24].

Males have a higher incidence rate of SCD compared to the age-matched female population, which substantiate the assumption of protection by female gender. Our findings cannot be explained by a different risk profile. We believe that traditional risk factors may play only a minor role in young SCD victims, as many traditional risk factors are life-style related and often manifests later in life. Some investigators have suggested this protection to be caused by female endogenous estrogen [35]. The so-called “estrogen-effect” has been considered to have both long-term and rapid effects, through both an atheroprotective effect on serum lipid concentrations [36–38] and through a direct action on blood vessels. The latter estrogen effect involves increasing vasodilatation and inhibiting response to blood vessel injury and the development of atherosclerosis [39, 40]. Another recently published study on estrogen treated male mice, found that these mice had increased mean lifespan [41]. On the other hand, researchers have suggested that male sex hormones decrease life expectancy in men. A previous study on human eunuchs (castrated males) found that their lifespan was 14 years longer than the non-castrated males [42]. Based on our findings, the female gender-advantage seems not to be limited to CAD, but also to arrhythmic events in other cardiac disease, both cardiomyopathies and primary arrhytmogenic diseases. While our study doesn’t contain concrete parameters to evaluate this hypothesis further, our finding that young women in their reproductive years have a gender-advantage, merits further investigation.

### Limitations

It is a limitation of the study that it was retrospective. Autopsy was not performed in all cases of sudden death, and we did not have access to family history of SCD in young age.

Due to the study design we did not have reliable data on traditional (life-style associated) risk factors associated with heart disease on all patients. This however can probably not explain the observed gender differences in young SCD. Furthermore, we have not been able to account for hormonal levels/changes in this retrospective design.

## Conclusions

Even though the incidence rate of SCD in young men is twice than that for women, causes of death is largely comparable and the huge difference in incidence rate is not explained by a different risk profile. SCD due to potentially inherited cardiac diseases is less often in women than in men, substantiating the assumption of protection by female gender. This merits further investigations.
